# DeepKinomeWeb: a quantitative, panel-level platform for kinase inhibitor screening and selectivity profiling

**DOI:** 10.1093/nar/gkag393

**Published:** 2026-04-29

**Authors:** Jisu Eun, Yeeun Lee, Seunghoon Yang, Donghwan Choi, Hyeonsu Na, Hyeyun Cho, Seungyoon Nam, Jinhyuk Lee

**Affiliations:** Bio-design Editing Research Center, Korea Research Institute of Bioscience and Biotechnology (KRIBB), Daejeon 34141, Korea; Department of Genome Medicine and Science, Gachon Institute of Genome Medicine and Science, Gachon University Gil Medical Center, Gachon University College of Medicine, Incheon 21565, Korea; Bio-design Editing Research Center, Korea Research Institute of Bioscience and Biotechnology (KRIBB), Daejeon 34141, Korea; Department of Bioinformatics, KRIBB School of Bioscience, University of Science and Technology (UST), Daejeon 34141, Korea; Bio-design Editing Research Center, Korea Research Institute of Bioscience and Biotechnology (KRIBB), Daejeon 34141, Korea; Bio-design Editing Research Center, Korea Research Institute of Bioscience and Biotechnology (KRIBB), Daejeon 34141, Korea; Department of Bioinformatics, KRIBB School of Bioscience, University of Science and Technology (UST), Daejeon 34141, Korea; Bio-design Editing Research Center, Korea Research Institute of Bioscience and Biotechnology (KRIBB), Daejeon 34141, Korea; Department of Bioinformatics, KRIBB School of Bioscience, University of Science and Technology (UST), Daejeon 34141, Korea; Department of Genome Medicine and Science, Gachon Institute of Genome Medicine and Science, Gachon University Gil Medical Center, Gachon University College of Medicine, Incheon 21565, Korea; Department of Health Sciences and Technology, Department of Translational-Clinical Medicine, Gachon Advanced Institute for Health Sciences and Technology (GAIHST), Gachon University, Incheon 21999, Korea; Bio-design Editing Research Center, Korea Research Institute of Bioscience and Biotechnology (KRIBB), Daejeon 34141, Korea; Department of Bioinformatics, KRIBB School of Bioscience, University of Science and Technology (UST), Daejeon 34141, Korea

## Abstract

Protein kinases are central targets in drug discovery, yet early-stage development of potent and selective inhibitors remains challenging due to high experimental costs and limited interpretability of large-scale screening data. Here, we present DeepKinomeWeb, an integrated web-based platform that transforms competition-based high-throughput screening data into actionable insights for kinase inhibitor prioritization. Built upon our previously validated deep learning regression model, DeepKinome, the platform enables quantitative prediction of kinase–inhibitor binding affinities and provides panel-level visualization of selectivity landscapes, selectivity metric calculations, and integrated structural and physicochemical analyses. Through its user-friendly interface, DeepKinomeWeb supports rational, data-driven decision-making for biologists and medicinal chemists, lowering the barrier to systematic selectivity assessment in kinase inhibitor discovery. DeepKinomeWeb is freely available to all users without any login requirement at https://str.kribb.re.kr/deepkinome.

## Graphical Abstract

**Graphical Abstract:**
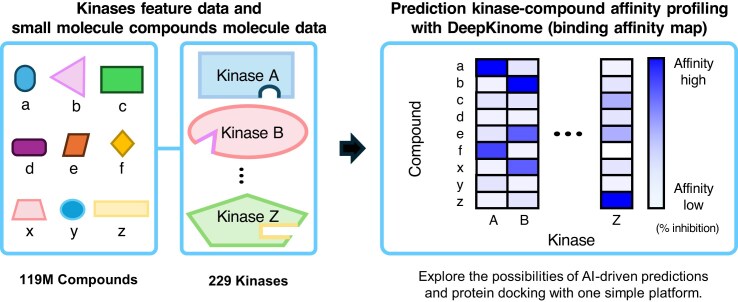

## Introduction

Protein kinases constitute one of the largest enzyme families in the human genome and serve as pivotal regulators of nearly all cellular functions, making them one of the most critical classes of drug targets in modern oncology [1, 2]. A primary challenge in the early stages of kinase drug discovery is the efficient identification of potent and selective inhibitors from competition-based high-throughput screening (HTS) campaigns. In these screens, systematic profiling across expansive kinase panels is essential to accurately assess a compound’s selectivity profile [3–5]. However, traditional interpretation of HTS results frequently relies on simplified hit-calling practices and narrow kinase-by-kinase inspection. Rather than examining individual kinases in isolation, it is important to view global selectivity patterns across a kinase panel; otherwise, consistently comparing multiple hits and anticipating off-target liabilities that may complicate downstream development becomes challenging.

The current landscape of computational tools designed for kinase inhibitor analysis reflects significant gaps in addressing these challenges. Specifically, three critical gaps persist. First, few computational tools support systematic screening across a broad kinase panel, limiting the ability to evaluate compound activity at the panel level. Second, there is a lack of integrated systems that enable users to assess and manage off-target liabilities within a single analytical framework. Third, although numerous AI-driven methods have been developed for drug–target binding prediction [6, 7], most predict only the presence or absence of an interaction, which limits meaningful prioritization of predicted target proteins for a given compound across a kinase panel. Representative examples include DeepDTA [8] and GraphDTA [9], which predict continuous binding affinity values but are available only as command-line tools without user-friendly web interfaces and do not provide panel-level selectivity profiling or integrated physicochemical analysis. DTI-MLCD [10] further exemplifies this limitation, as it is based on multi-label classification and remains within the scope of binary interaction prediction. KLIFS [11] provides valuable structural information on kinase–ligand interactions, but it is not designed to support integrated panel-level selectivity assessment and compound prioritization within a single workflow. Despite substantial efforts devoted to studying kinase–drug interactions throughout the drug development process, these challenges remain largely unaddressed. Currently, individual tools for binding affinity prediction, structure-based docking [12], ADMET profiling, and selectivity assessment are available; however, freely accessible web services that integrate these analyses into a unified workflow for panel-level hit evaluation remain scarce. Collectively, these gaps highlight the need for an integrated, user-friendly platform that supports quantitative prediction, panel-level selectivity assessment, and interpretable downstream analysis.

DeepKinomeWeb was developed to translate the DeepKinome model [10] into a practical, researcher-friendly web service that addresses these needs. By combining data-driven deep learning prediction with structure-based docking analysis, ADMET profiling, selectivity index (SI) profiling, and external resource links, DeepKinomeWeb enables users to cross-validate predicted kinase interactions from complementary analytical perspectives and to prioritize candidate compounds more rationally for downstream experimental validation.

## Materials and methods

### Design and implementation

This section describes the core components of DeepKinomeWeb. The platform is built upon a validated deep learning model and integrates a newly developed, user-centered system architecture with analytical modules that enable data-driven evaluation of binding characteristics and selectivity for user-submitted compounds.

#### The DeepKinome predictive engine: a foundation of validated performance

**Figure 1.**
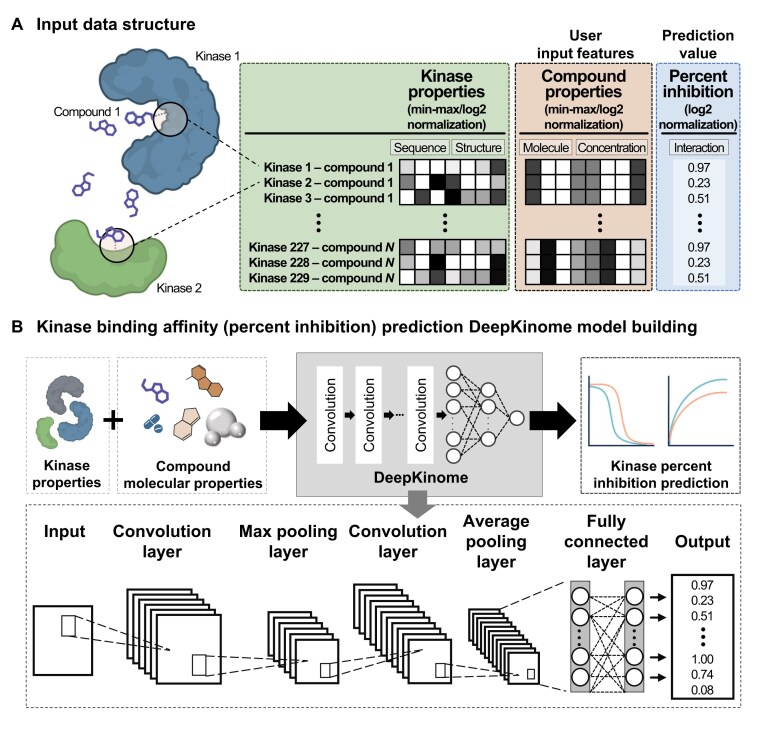
Architecture of the DeepKinome framework for kinase–compound binding affinity prediction. (**A**) Input data structure used in DeepKinome, where kinase properties derived from sequence and structural features and compound properties calculated from molecular descriptors are normalized and combined to represent kinase–compound interaction pairs for model input. (**B**) DeepKinome model architecture for kinase binding affinity prediction, consisting of multiple convolutional layers followed by fully connected neural network layers that learn interaction patterns between kinase and compound features, ultimately producing quantitative percent inhibition predictions for each kinase–compound pair. Adapted from Lee *et al*. (2025), “DeepKinome: quantitative prediction of kinase binding affinity by a compound using a deep learning-based regression model” [doi: 10.3389/fmolb.2025.1698891] [13], with permission under the Creative Commons Attribution 4.0 International License (CC BY 4.0).

The deep learning engine of DeepKinomeWeb is based on our previously published DeepKinome model, a 20-layer convolutional neural network-based deep learning regression model designed to quantitatively predict kinase–inhibitor binding affinity [13]. The model was trained and validated on a large dataset of competition-based HTS data (KINOMEscan) from the Library of Integrated Network-Based Cellular Signatures (LINCS) project (Fig. 1A).

The model architecture uses two sets of input features: 8551 features representing the kinase, derived from amino acid sequence, secondary structure, and active site properties; and 2757 features representing the small-molecule compound, calculated as chemical descriptors from its two-dimensional structure (Fig. 1B). The model produces a continuous prediction of percent inhibition (% inhibition), a metric widely used as a surrogate for the quantitative binding affinity in large-scale screening assays. In the KINOMEscan assay, percent inhibition reflects the degree of competitive displacement of a reference ligand from the kinase binding site. Values closer to 0% indicate stronger compound–kinase binding due to more effective ligand displacement, whereas values closer to 100% indicate weaker interactions.

In our previous report [13], DeepKinome was rigorously benchmarked against nine other AI models and demonstrated superior predictive performance, achieving a root mean square error of 1.157, a Pearson’s correlation coefficient of 0.743, and a coefficient of determination (*R*²) of 0.535 on the L1000 test dataset. In addition, the performance improvement of DeepKinome over competing models was statistically validated through three-fold cross-validation (three-fold CV) with Welch’s *t*-test, showing significant differences across evaluation metrics (*P* < .01).

#### From model to platform: the DeepKinomeWeb architecture and enhancements

**Figure 2.**
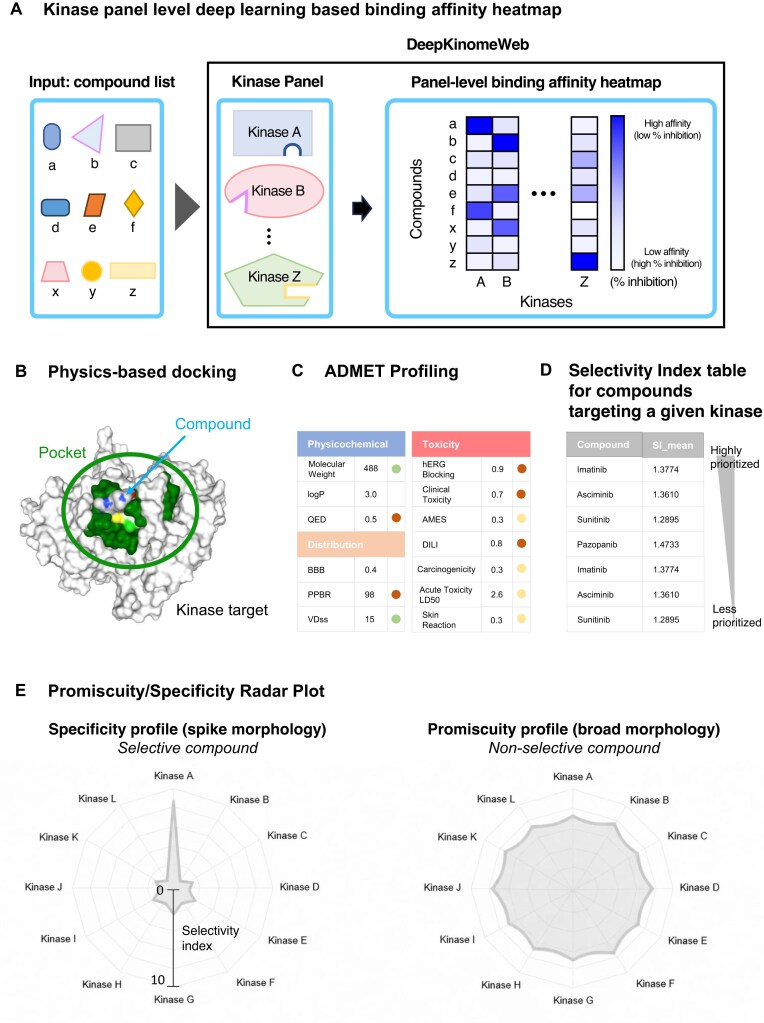
Main analytical outputs of the DeepKinomeWeb platform. (**A**) Overview of the DeepKinomeWeb workflow. A list of input compounds is processed by the DeepKinome predictive engine in conjunction with a kinase panel, producing a panel-level binding affinity heatmap as output. The heatmap represents predicted percent inhibition values across multiple kinases, enabling systematic evaluation of compound–kinase interactions. (**B**) Physics-based docking visualization of a compound within a kinase structure, displayed when the docking procedure is enabled. The predicted binding pocket is highlighted, and the compound is positioned within the kinase target to illustrate potential structural interactions. (**C**) ADMET profiling results for a compound. Physicochemical properties, toxicity, and distribution-related features are summarized to support evaluation of drug-like properties. (**D**) SI table for compounds within a given kinase. Compounds are ranked based on their SI, allowing prioritization from highly selective to less selective candidates. (**E**) Promiscuity/specificity radar plot. Each axis represents an individual kinase, and the radial distance (radius) corresponds to the SI value for that kinase. The numerical values shown along the radial scale (e.g., 0–10) indicate the magnitude of the SI, where larger values reflect higher selectivity. For a given compound, SI values across kinases are plotted as vertices on each axis and connected to form a polygon. The shaded (gray) region represents this polygon and is filled to improve visibility of the overall selectivity pattern. Selective compounds exhibit sharp, spike-like morphologies, indicating high preference toward specific kinases, whereas non-selective compounds display broader, more uniform shapes, reflecting promiscuous interactions across the kinase panel.

DeepKinomeWeb extends the standalone DeepKinome model by integrating its predictions with a set of analytical tools within a unified web-based environment. Rather than serving solely as a deployment interface, the platform provides an integrated workflow that supports downstream analysis of prediction results (Fig. 2).

The main features of the platform are summarized below:

- Quantitative binding affinity prediction: The server provides continuous affinity scores (percent inhibition), moving beyond simplistic binary (bind/non-bind) classifications. These predictions are visualized as a heatmap, where each cell represents the predicted interaction between a compound and a kinase. Lower values (stronger binding) and higher values (weaker binding) can be directly compared across the kinase panel. This representation enables intuitive assessment of both on-target potency and off-target interactions, allowing users to rapidly identify compounds with strong target binding and minimal off-target effects.
- Selectivity index: The platform computes quantitative SI metrics to summarize the relative balance between on-target and off-target percent inhibition for each compound. Based on predicted percent inhibition values across a kinase panel, two complementary indices are defined. SI_mean_ is calculated as:
  (1) \begin{eqnarray*}
{\mathrm{S}}{{{\mathrm{I}}}_{\textrm{mean}}}{\mathrm{\ }} = {\mathrm{\ }}\frac{\mathrm{ {G{{M}}_{\mathrm{ off}}}}}{{{{\mathrm{ I}}_{\mathrm{ on }- \textrm{target}}}}},
\end{eqnarray*}

where $\mathrm{ G{{M}_{{\mathrm{off}}}}}$ denotes the geometric mean of percent inhibition values across all off-target kinases and $\mathrm{ {{I}_{on - \textrm{target}}}}$ denotes the percent inhibition of the intended on-target kinase. This metric provides a global measure of selectivity across the kinase panel. SI_worst_ is defined as:


(2) \begin{eqnarray*}
\mathrm{ S{{I}_{\textrm{worst}}} = \frac{{min\left( {{{I}_{off - \textrm{targets}}}} \right)}}{{{{I}_{on - \textrm{target}}}}}},
\end{eqnarray*}

where $\mathrm{ {\mathrm{min}}( {{{I}_{off - \textrm{targets}}}} )}$ represents the lowest percent inhibition among off-target interactions. This metric captures the most critical off-target liability. Because lower percent inhibition values correspond to stronger compound–kinase binding, higher SI values indicate better selectivity, meaning that off-target interactions are weaker relative to the intended target.

- Integrated structure-based docking analysis: To provide complementary structural insights, the server integrates traditional docking simulations using AutoDock Vina [14] alongside AI-driven predictions. The AutoDock Vina workflow is described in Supplementary Note S1. This module generates structurally plausible binding poses and consistent docking affinity estimates that complement AI-based affinity predictions.
- Physicochemical and ADMET-related property profiling: The platform calculates and displays key molecular descriptors and other properties related to absorption, distribution, metabolism, excretion, and toxicity (ADMET) using the ADMET-AI platform [15]. This information supports an early-stage assessment of a compound’s development potential.

#### From model to platform: the DeepKinomeWeb architecture and enhancements

The DeepKinomeWeb platform is built with modern web technologies to ensure a responsive and reliable user experience. The frontend is implemented using Next.js, built on React and JavaScript, and utilizes the NGL Viewer library [16] for interactive 3D visualization of molecular structures. The backend is built on the Python FastAPI framework, deployed with a Unicorn and Gunicorn server configuration, which handles user requests and data processing. Job submissions are managed by a Python-based job management system utilizing semaphores and locking mechanisms, which schedules and executes the computationally intensive tasks of prediction, analysis, and docking. This architecture ensures that user-submitted jobs are processed efficiently without requiring computational power from the client side.

## Results

### Web server usage and workflow

The DeepKinomeWeb workflow is designed to be intuitive and accessible, guiding a user from a single compound of interest to a comprehensive, multi-faceted analysis report. The entire process is automated, requiring minimal user input to generate a rich set of results for experimental prioritization.

#### Input submission

**Figure 3.**
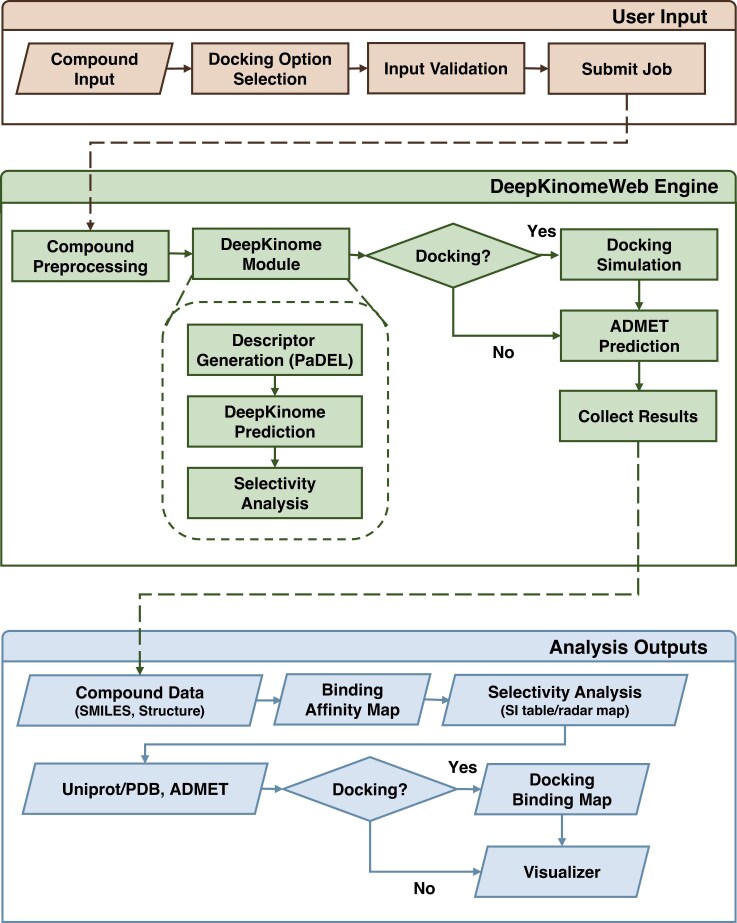
Workflow of the DeepKinomeWeb platform showing user input, DeepKinomeWeb engine processing, and analysis outputs. Submitted compounds undergo preprocessing, affinity prediction, selectivity analysis, optional docking simulation, and ADMET prediction, and the platform generates binding affinity maps, selectivity analyses, ADMET results, and optional docking-based structural visualizations.

In the topmost stage of the workflow shown in Fig. 3, users initiate an analysis by submitting a small-molecule compound through the web-based input interface. Compounds can be provided either by entering a PubChem compound identifier (CID) [17] or by uploading a structure file in SDF format. Submitted compounds are automatically screened against a predefined kinase panel consisting of 229 kinases derived from the DeepKinome training dataset. The detailed list of the 229 kinases is provided in Supplementary Table S1.

Before execution, users may optionally enable docking simulation through the docking option selection, allowing either prediction-only analysis or combined prediction and docking workflows. The server then performs automatic input validation to ensure that compound structures and job parameters are correctly formatted and suitable for analysis. Once validation is completed, the analysis job is submitted to the server queue and processed automatically.

The server also provides a sample data loading option, allowing users to explore the platform workflow and examine a pre-computed example report without requiring prior input.

AI-based prediction currently supports up to 50 compounds per run. However, due to additional computational demands, enabling docking simulations reduces this limit to 10 compounds per run. For larger compound sets, users may first perform prediction-only screening and subsequently select top-ranked compounds for docking analysis. This two-step usage strategy enables efficient resource utilization while still allowing detailed structural evaluation of prioritized candidates.

#### Server-side processing workflow

In the middle stage of the workflow shown in Fig. 3, the DeepKinomeWeb server automatically performs a sequence of processing steps to generate prediction and analysis results.

First, compound structures are standardized according to the input type. When compounds are submitted using PubChem CIDs, corresponding SDF structure files are automatically retrieved from the database. For user-uploaded files, the server validates whether the provided files are correctly formatted SDF structures. After validation, compound lists are organized, SMILES strings are extracted, and 2D structural representations are generated for later display.

Next, molecular descriptors required for prediction are generated using the PaDEL descriptor tool [18]. These descriptors are then used as input for the DeepKinome prediction module, which estimates kinase–compound binding affinities across the kinase panel. Based on predicted percent inhibition values, selectivity analysis is subsequently performed, and both SI_mean_ and SI_worst_ metrics are computed to quantify compound selectivity.

If docking simulation is enabled during input submission, compound–kinase pairs are processed using AutoDock Vina to compute docking-based binding affinity scores. These scores are aggregated to generate an AutoDock Vina affinity heatmap, providing a structure-based complement to AI-predicted binding profiles. The resulting docking poses are also retained and later visualized through an interactive molecular viewer, allowing users to inspect predicted protein–ligand interactions. If docking is not requested, this step is skipped to reduce computational cost.

In parallel, physicochemical and pharmacokinetic properties are predicted using the ADMET-AI model, providing early insight into absorption, distribution, metabolism, excretion, and toxicity characteristics of candidate compounds.

Finally, outputs generated from all modules are aggregated and organized into a result package, which is transferred to directories linked to the web interface, enabling immediate visualization and interactive exploration on the results page.

#### Output and interpretation

In the final stage of the workflow shown in Fig. 3, results are presented on a single interactive page composed of multiple panels, each providing complementary information to support compound evaluation and prioritization.

- Input data overview: The results page first summarizes the submitted compounds, including compound names (when available), SMILES strings, and generated structural representations. This overview allows users to verify input compounds before proceeding with further analysis.
- Binding affinity map: The primary visualization is a heatmap displaying predicted percent inhibition values for each compound across the kinase panel. Color intensity corresponds to predicted binding strength, with lower percent inhibition values indicating stronger interactions. This global view enables rapid identification of potent on-target interactions and potential off-target effects. Additionally, users can refer to external resources through provided links for further information.
- Selectivity analysis: This panel provides quantitative selectivity evaluation through two complementary analysis modes. The first mode is designed for researchers who have identified a primary on-target kinase and wish to screen a compound library for the most promising candidates. Users specify an on-target kinase, and the system calculates two SIs (SI_mean_ and SI_worst_) for each compound. Compounds exhibiting strong inhibition of the on-target and weak inhibition of off-target kinases are prioritized as high-quality hits. The second mode provides a comprehensive binding profile for a single compound across the human kinome to identify potential off-target liabilities or repurposing opportunities. Users input a compound, which is evaluated against an integrated panel of 229 kinases derived from the LINCS KINOMEscan dataset. The platform generates a promiscuity/specificity radar plot, highlighting targets that are effectively inhibited by the compound. This systematic profiling is essential for uncovering latent polypharmacology and predicting mechanisms of action.
- Protein annotation and ADMET information: To provide biological and pharmacological context, the platform displays protein annotations retrieved from UniProt [19], structural metadata from the Protein Data Bank (PDB) [20], and predicted physicochemical and ADMET-related properties of the compound. These modules support interpretation of prediction results and early assessment of compound developability.
- Docking-based structural visualization: When docking simulation has been performed, AutoDock Vina affinity results are presented alongside an interactive molecular viewer implemented using NGL Viewer. This viewer displays predicted compound binding poses within kinase structures and provides pocket-level structural information for inspection of protein–ligand interactions. When docking is not executed, the viewer still allows visualization of the protein structure, although compound binding poses are not available.

The interactive design of the results interface allows users to inspect heatmap elements, sort selectivity tables, explore radar plots, and manipulate 3D structures. This integrated presentation enables holistic interpretation of prediction, selectivity, structural, and pharmacological data, facilitating informed compound prioritization decisions.

#### Case study: comparative evaluation of kinase inhibitor selectivity using DeepKinomeWeb

To assess whether DeepKinomeWeb can support selectivity-guided prioritization of kinase inhibitors, we analyzed three FDA-approved ATP-competitive kinase inhibitors that were absent from the LINCS training dataset: brigatinib (PubChem CID: 68165256), a highly selective ALK inhibitor with a Type I binding mode [21, 22]; ponatinib (PubChem CID: 24826799), a multi-target inhibitor with a Type II binding mode [23, 24]; and sunitinib (PubChem CID: 5329102), a broad-spectrum multi-kinase inhibitor reported to exhibit both Type I and Type II binding characteristics [25, 26]. These compounds were selected from the KLIFS database [11] to represent a spectrum of selectivity profiles, from highly selective to broad-spectrum. A detailed description of kinase inhibitor binding types (Type I–II) is provided in Supplementary Note S2.

**Figure 4.**
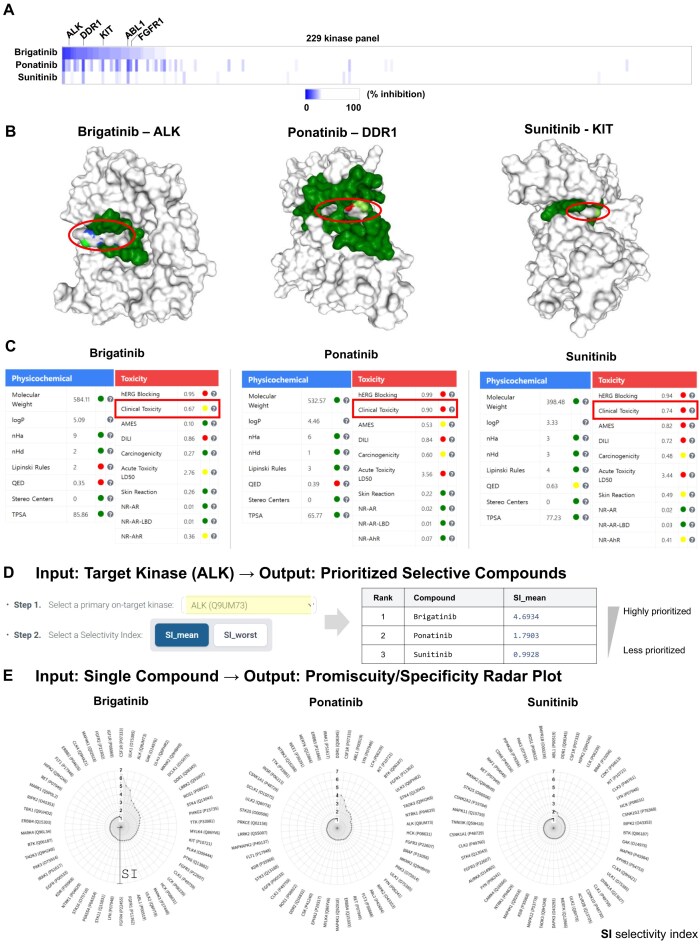
Case study using three FDA-approved ATP-competitive kinase inhibitors absent from the LINCS training dataset: brigatinib (Type I, selective ALK inhibitor), ponatinib (Type II, multi-target inhibitor), and sunitinib (Type I/II, broad-spectrum multi-kinase inhibitor). (**A**) Binding affinity heatmap across the 229-kinase panel. Each cell represents a compound–kinase pair; darker blue indicates lower percent inhibition (stronger predicted binding). (**B**) Predicted docking poses generated by the integrated AutoDock Vina module for brigatinib–ALK, ponatinib–DDR1, and sunitinib–KIT. Kinase structures are shown in white surface representation. Predicted binding pockets are highlighted in green. Docked compounds are colored by atom type: carbon (gray), oxygen (red), nitrogen (blue), and fluorine (light green). Red circles indicate the predicted binding site of each compound. (**C**) Predicted physicochemical and ADMET properties. Clinical toxicity scores are highlighted (red boxes) for comparison across compounds. (**D**) Kinase-based selectivity analysis ranking compounds by SI_mean_ for ALK. (**E**) Compound-based promiscuity/specificity radar plots. Each axis represents a kinase, and radial distance corresponds to the SI_mean_ value.

The binding affinity map (Fig. 4A) displays predicted percent inhibition values for the three compounds across the 229-kinase panel. For visualization, kinases annotated as targets for each compound in the KLIFS database [11] and ranked within the top 10 by DeepKinome-predicted percent inhibition were labeled in the heatmap. When sorted by ascending percent inhibition using brigatinib as the reference, the top-ranking kinases corresponded well to the known target profiles: ALK was highly ranked for brigatinib, KIT for sunitinib, and multiple ponatinib-associated kinases including DDR1, ABL1, KIT, and FGFR1 were enriched near the top. Brigatinib displayed the lowest predicted percent inhibition for ALK among the three compounds, indicating the strongest predicted on-target interaction and the most selective binding pattern.

The integrated AutoDock Vina module generated predicted binding poses for each compound–kinase pair (Fig. 4B). The predicted pose of brigatinib occupies the ATP-binding site of ALK (PDB: 4Z55; pocket volume: 870 Å³; 19 residues), consistent with a Type I binding mode. The predicted pose of ponatinib spans both the ATP site and an adjacent allosteric region of DDR1 (PDB: 6FEX; pocket volume: 4583 Å³; 60 residues), consistent with a Type II binding mode. The predicted pose of sunitinib adopts a Type II binding orientation in KIT (PDB: 4HVS; pocket volume: 1330 Å³; 29 residues). These structural observations are consistent with the known binding modes of each inhibitor.

Predicted physicochemical and ADMET properties provided a complementary perspective on compound evaluation (Fig. 4C). Based on molecular weight and logP, sunitinib showed the most favorable physicochemical profile (MW = 398.48; logP = 3.30), followed by ponatinib (MW = 532.57; logP = 4.46) and brigatinib (MW = 584.11; logP = 5.09). However, the predicted toxicity profile showed a different ranking: ponatinib exhibited the highest predicted clinical toxicity score (0.90), consistent with its reported cardiovascular adverse events [27], while brigatinib showed the lowest score (0.67), in agreement with its clinically favorable safety profile [28]. Sunitinib ranked between the two (0.74). This difference illustrates that physicochemical favorability does not necessarily correlate with safety, underscoring the value of integrated ADMET profiling for early-stage decision-making.

The kinase-based selectivity analysis (Fig. 4D) quantitatively ranked the three compounds by their selectivity toward ALK. Brigatinib ranked first (SI_mean_ = 4.6934), followed by ponatinib (1.7903) and sunitinib (0.9928), reflecting decreasing selectivity toward ALK.

The compound-based promiscuity/specificity radar plots (Fig. 4E) further visualized selectivity differences at the whole-panel level. Brigatinib displayed a sharp, spike-like profile indicative of high selectivity, whereas ponatinib and sunitinib exhibited broader, more rounded profiles consistent with multi-kinase interaction patterns.

#### Statistical validation of selectivity Index and comparison of DeepKinomeWeb with docking

**Figure 5.**
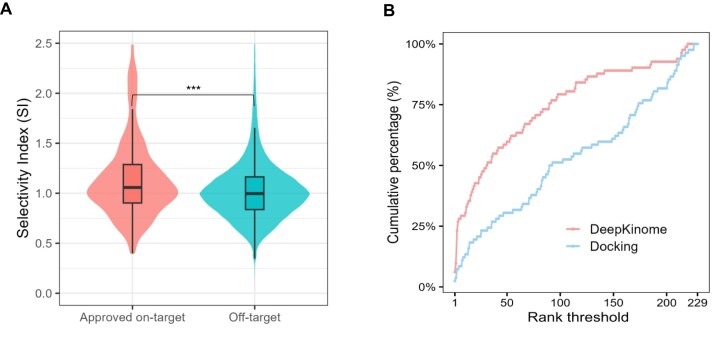
Statistical validation of the SI and comparison of DeepKinomeWeb with docking. (**A**) Violin plot comparing SI_mean_ between FDA-approved on-target (red) and off-target (blue). FDA-approved on-target shows a slight upward shift in SI_mean_ (*n* = 422; median = 1.058) relative to off-target (*n* = 154 480; median = 0.997). Although the median difference is small, the distributions are statistically distinguishable (Wilcoxon *P* = 2.48 × 10^−9^) with substantial overlap. (**B**) Cumulative rank curve comparing how effectively DeepKinome and AutoDock Vina place known target kinases near the top of the ranked list for 82 FDA-approved compounds whose approved targets are included in the 229-kinase DeepKinomeWeb panel. For each compound, all 229 kinases were ranked according to the corresponding score, and the *x*-axis indicates the rank range (1–229). The *y*-axis represents the cumulative percentage of compounds for which the approved target kinase is included within the given rank threshold. When multiple approved target kinases were annotated for a single compound, only the highest-ranked target was used as the representative target. Because all kinases are included at *x* = 229, the curve reaches 100% at that point. A higher curve indicates that the method places the true target kinase at a higher rank.

We next evaluated whether SI_mean_ captures on-target preference at the population level by comparing FDA-approved on-target compounds and off-target compounds. Detailed definitions of the reference datasets are provided in Supplementary Note S3. Briefly, we first identified 82 FDA-approved compounds whose approved target kinases are represented in the 229-kinase DeepKinomeWeb panel. Because individual compounds can have multiple approved kinase targets, these compounds yielded 422 FDA-approved on-target compound–kinase pairs after DeepKinome processing. Off-target pairs were constructed from predicted binding affinity data between the corresponding drugs and non-annotated kinases within the DeepKinomeWeb panel. As shown in Fig. 5A, FDA-approved on-target pairs exhibited a modest upward shift in SI_mean_ relative to off-target pairs, indicating greater on-target preference at the population level. Although the separation was modest and the two distributions still showed substantial overlap, the difference remained statistically significant, suggesting that SI_mean_ is associated with FDA-approved on-target status. Similar distributional tendencies were also observed in additional reference-group comparisons, including non-kinase proteins, general compounds, and unapproved on-target kinases (Supplementary Fig. S1 and  Supplementary Note S3). Collectively, these results suggest that SI_mean_ captures biologically relevant target preference at the population level and is best interpreted as a comparative prioritization metric rather than as a single universal cutoff.

Next, using these 82 FDA-approved compounds, we directly compared the prioritization performance of DeepKinome and conventional docking. Specifically, we asked how effectively each method places true target kinases near the top of the ranked list for a given compound across the 229-kinase panel, thereby assessing the practical utility of AI-based binding affinity prediction relative to physics-based docking. For each compound, all 229 kinases were ranked according to either DeepKinome-predicted percent inhibition or AutoDock Vina docking score. When multiple approved target kinases were annotated for a single compound, only the highest-ranked target was retained as the representative target. DeepKinome placed the true approved target kinase in higher-ranking positions more frequently than AutoDock Vina across most of the ranking range, and its cumulative curve remained consistently higher overall (Fig. 5B). These results suggest that DeepKinome can serve as a more effective first-pass screening tool for kinase target prioritization, whereas docking may be more appropriately interpreted as a complementary structure-based follow-up analysis.

## Discussion

In this study, we present DeepKinomeWeb and demonstrate its utility through a series of complementary analyses. Using a case study of three FDA-approved ATP-competitive kinase inhibitors (brigatinib, ponatinib, and sunitinib) that were absent from the LINCS training dataset, we show that the platform distinguishes compounds with distinct selectivity profiles—ranging from highly selective to broad-spectrum—through its integrated workflow, including binding affinity prediction, docking visualization, ADMET profiling, and selectivity analysis (Fig. 4). Statistical validation using DrugBank-derived reference sets further confirms that the platform’s SI metric (SI_mean_) captures biologically relevant on-target preferences at the population level (Fig. 5A). In addition, cumulative ranking comparisons demonstrate that DeepKinome consistently recovers approved on-target kinases at higher ranks than AutoDock Vina alone (Fig. 5B), supporting the use of data-driven affinity prediction as an effective first-pass prioritization step complementary to physics-based docking. By providing an integrated platform for quantitative prediction, selectivity profiling, and structural analysis, DeepKinomeWeb can assist researchers in navigating the chemical and biological space inherent to early-stage drug discovery.

The utility of DeepKinomeWeb is best understood in the context of other available computational tools. Several computational resources address related but distinct aspects of kinase inhibitor analysis. General target prediction servers such as SuperPred 3.0 [29] provide drug classification and target identification but do not offer quantitative binding affinity estimates. Kinase-focused databases such as KinaseMD [30] and the recently published Dr. Kinase [31] catalog known mutation–drug resistance relationships; however, they do not predict activity for novel compounds. Structure-based tools, including AlphaFold 3 [32] and DiffDock [33], generate binding poses but are computationally prohibitive for panel-level screening across hundreds of kinases. DeepKinomeWeb occupies a distinct niche by combining quantitative, regression-based affinity prediction across a 229-kinase panel with integrated selectivity metrics, docking, and ADMET profiling in a single, user-friendly interface. Notably, unlike existing tools that typically evaluate a single compound against a single target, DeepKinomeWeb supports simultaneous screening of up to 50 compounds across the full 229-kinase panel within a single analysis run, enabling systematic selectivity comparison that is essential for early-stage hit prioritization. A detailed comparison of DeepKinomeWeb with related platforms is provided in Supplementary Table S2.

Several limitations of the current platform should be noted. The human genome encodes approximately 518 protein kinases [1]; however, the DeepKinomeWeb panel currently covers 229 kinases, as the DeepKinome model was trained exclusively on kinases for which both experimentally determined protein structures in the Protein Data Bank and quantitative percent inhibition data from the LINCS KINOMEscan dataset were available. Consequently, kinases lacking experimental structural data or standardized binding assay measurements are not represented in the current panel, which may limit the platform’s coverage of therapeutically relevant targets.

Future development will focus on expanding kinase coverage. Approximately 100 additional kinases with available KINOMEscan measurements were excluded from the current training set due to the absence of experimentally determined crystal structures. Leveraging protein structure prediction methods such as AlphaFold [32] to generate reliable structural models for these kinases would enable their incorporation into the panel. Integration of complementary bioactivity data sources, such as PubChem BioAssay [17], may further broaden the predictive scope by enabling cross-platform validation and enriching the diversity of training data.

In addition, certain proteins such as pyruvate kinase M2 are classified as “kinases” due to their phosphotransferase activity but belong to distinct enzyme families outside the canonical human protein kinase superfamily [1, 34]. Because such kinase-like proteins are not represented in the current KINOMEscan-derived panel, their inclusion in future versions could enhance the platform’s applicability to a broader range of drug targets.

In conclusion, DeepKinomeWeb aims to support the transition of deep learning from a pure prediction technology to a practical decision-support tool. It provides the drug discovery community with an accessible, freely available platform that may help streamline the identification of candidate kinase inhibitors and de-risk downstream experimental testing in a resource-efficient manner.

## Supplementary Material

gkag393_Supplemental_File

## Data Availability

DeepKinomeWeb is freely accessible without login at https://str.kribb.re.kr/deepkinome. The source code is available at https://github.com/labnams/DeepKinome and is permanently archived at Zenodo (DOI: 10.5281/zenodo.18501372). Example input data and precomputed results are available within the web server interface. Prediction results are generated dynamically upon user submission.
