# Supplementary material for: DeepKinomeWeb: a quantitative, panel-level platform for kinase inhibitor screening and selectivity profiling
Source: Nucleic Acids Res. 2026 Apr 29;54(W1):W246–56. doi: 10.1093/nar/gkag393 (PMC13355055; doi:10.1093/nar/gkag393)
Supplement: gkag393_Supplemental_File [file gkag393_supplemental_file.pdf]

SUPPLEMENTARY DATA

DeepKinomeWeb: A Quantitative, Panel-Level Platform for Kinase Inhibitor Screening and Selectivity Profiling

AUTHORS

Jisu Eun[1,†], Yeeun Lee[2,†], Seunghoon Yang[1,3], Donghwan Choi[1], Hyeonsu Na[1,3], Hyeyun Cho[1,3], Seungyoon Nam[2,4,*] and Jinhyuk Lee[1,3,*]

[1] Bio-design Editing Research Center, Korea Research Institute of Bioscience and Biotechnology (KRIBB), Daejeon, 34141, Korea.

[2] Department of Genome Medicine and Science, Gachon Institute of Genome Medicine and Science, Gachon University Gil Medical Center, Gachon University College of Medicine, Incheon, 21565, Korea.

[3] Department of Bioinformatics, KRIBB School of Bioscience, University of Science and Technology (UST), Daejeon, 34141, Korea.

[4] Department of Health Sciences and Technology, Gachon Advanced Institute for Health Sciences and Technology (GAIHST), Gachon University, Incheon, 21999, Korea.

[†] Jisu Eun and Yeeun Lee have contributed equally to this work.

* To whom correspondence should be addressed. Email: nams@gachon.ac.kr

Correspondence may also be addressed to: jinhyuk@kribb.re.kr

Table of Contents

Supplementary Figure S1. Comparative analysis of SI_{mean} distributions…............................. 3

Supplementary Figure S2. Comparative analysis of SI_{worst} distributions................................ 4

Supplementary Note S1. AutoDock Vina workflow…….. 6

Supplementary Note S2. Classification of Kinase Inhibitor Binding Types…………………… 7

Supplementary Note S3. Test Data Description.. 8

Supplementary Note S4. Interpretation of Divergent Selectivity Patterns in FDA-Approved Kinase Inhibitors.. 9

Supplementary Table S1. Comparison of DeepKinomeWeb with existing computational platforms for kinase inhibitor analysis and drug–target interaction prediction...................... 10

Supplementary Table S2. Server 229 gene list and corresponding UniProt accessions…... 11

References..14

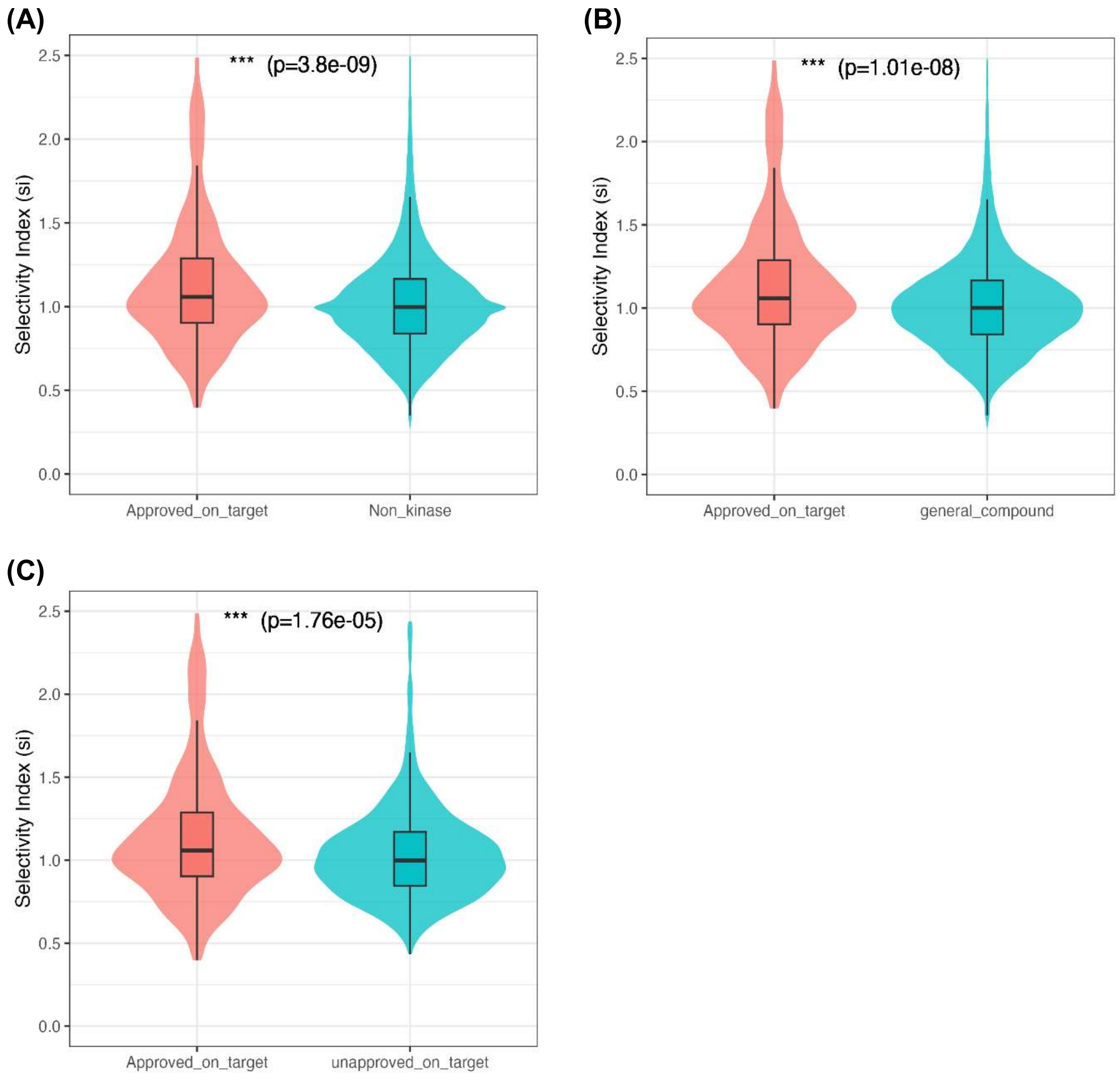

Supplementary Figure S1. Comparative analysis of SI_{mean} distributions.

(A) Violin plot comparing SI_{mean} between approved on-target (red) and non-kinase (blue) groups. Approved on-target kinases show a slight upward shift in SI_{mean} (n = 422; median = 1.058) relative to non-kinase proteins (n = 155,019; median = 0.998), indicating a tendency toward higher selectivity in approved on-target kinases. Although the median difference is small, the distributions are statistically distinguishable (Wilcoxon $p = 3.80 \times 10^{-9}$) with substantial overlap.

(B) Violin plot comparing SI_{mean} between approved on-target (red) and general compound (blue) groups. The approved on-target group (n = 422; median = 1.058) is modestly shifted toward higher SI_mean values compared with the general compound group (n = 104,811; median = 1.001). The distributions are statistically distinguishable (Wilcoxon $p = 1.01 \times 10^{-8}$) with substantial overlap.

(C) Violin plot comparing SI_{mean} between approved on-target (red) and unapproved on-target (blue) groups. Approved on-target kinases exhibit a slight upward shift in SI_mean (n = 422; median = 1.058) relative to unapproved on-target kinases (n = 585; median = 0.998), although the distributions largely overlap (Wilcoxon $p = 1.76 \times 10^{-5}$).

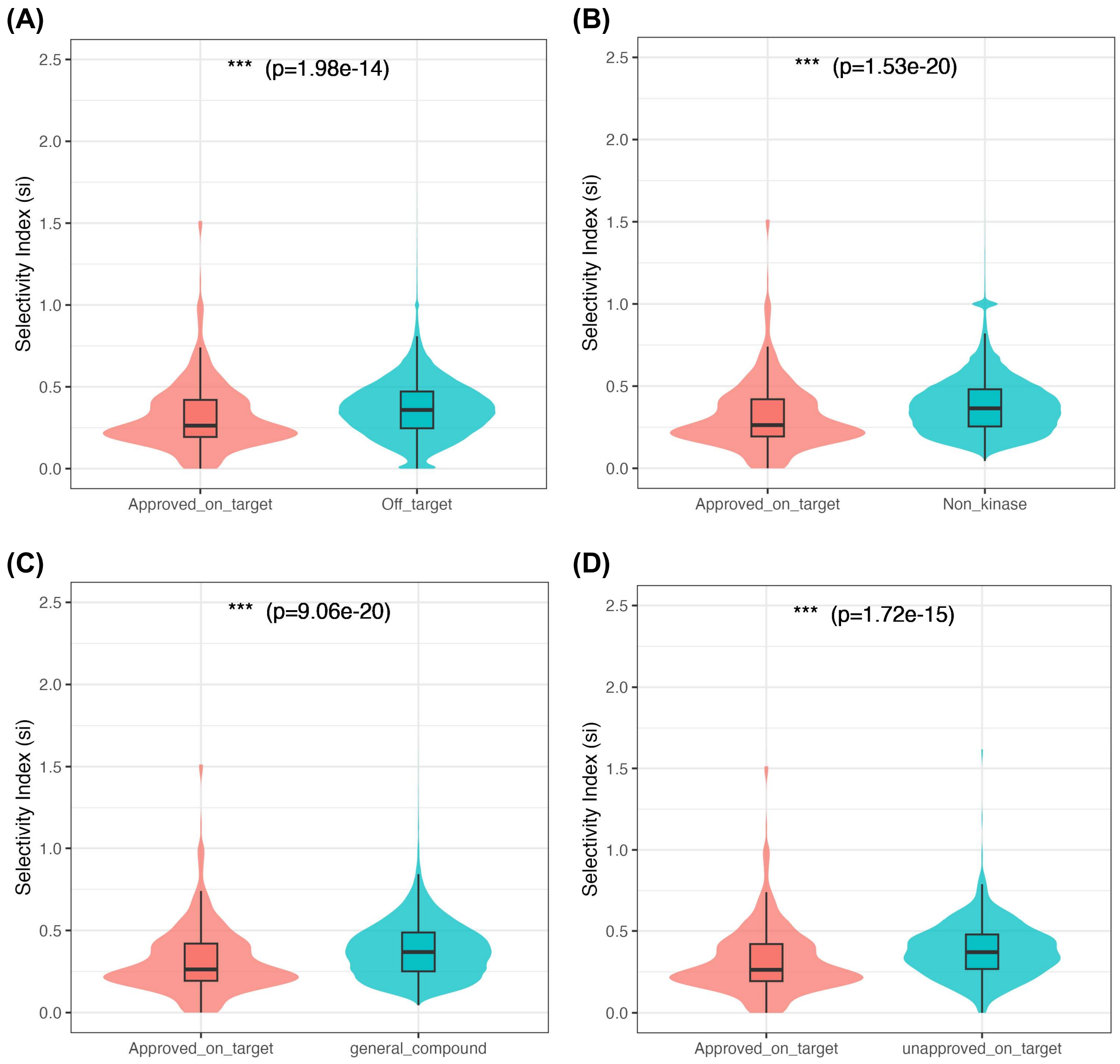

Supplementary Figure S2. Comparative analysis of SI_{worst} distributions.

(A) Violin plot comparing SI_{worst} between approved on-target (red) and off-target (blue) groups. The approved on-target group (n = 451; median = 0.263) is shifted toward lower SI_{worst} values compared to the off-target group (n = 156,201; median = 0.358). This suggests that SI_{worst} acts as a sensitive indicator for the risk posed by the most competitive off-target interactions. The distributions are statistically distinguishable (Wilcoxon $p = 1.98 \times 10^{-14}$).

(B) Violin plot comparing SI_{worst} between approved on-target (red) and non-kinase (blue) groups. The approved on-target group (n = 451; median = 0.263) exhibits a distinct distribution pattern compared to the non-kinase group (n = 156,636; median = 0.365). The distributions are statistically distinguishable (Wilcoxon $p = 1.53 \times 10^{-20}$), supporting the utility of SI_{worst} in capturing risk from strong binding partners.

(C) Violin plot comparing SI_{worst} between approved on-target (red) and general compound (blue) groups. The approved on-target group (n = 451; median = 0.263) shows a statistically distinct distribution compared to the general compound group (n = 105,798; median = 0.368). The distributions are clearly distinguishable (Wilcoxon $p = 9.06 \times 10^{-20}$), highlighting the metric's ability to identify “worst-case” binding scenarios.

(D) Violin plot comparing SI_{worst} between approved on-target (red) and unapproved on-target (blue) groups. Approved on-target kinases (n = 451; median = 0.263) show a statistically significant difference compared to unapproved on-target kinases (n = 877; median = 0.370). Although there is overlap, the distributions are distinguishable (Wilcoxon p = 1.72×10^{-15}), indicating the potential of SI_{worst} to detect subtle risk differences.

Supplementary Note S1. AutoDock Vina workflow

The AutoDock Vina docking workflow consists of the following steps: (i) automated identification of ligand-accessible surface pockets, followed by selection of the largest pocket corresponding to the canonical ATP-binding site of kinases; (ii) preparation of receptor and ligand structures in PDBQT format; (iii) docking using AutoDock Vina with uniform grid dimensions and exhaustiveness settings; and (iv) extraction of the lowest binding energy from cluster-representative conformations, where more negative (lower) affinity values indicate stronger binding.

Supplementary Note S2. Classification of Kinase Inhibitor Binding Types

Kinase inhibitors are classified based on the conformational state of the target kinase and the binding site they occupy. The most widely used classification distinguishes Type I and Type II inhibitors according to their interaction with the DFG (Asp-Phe-Gly) motif, a conserved regulatory element in the activation loop of protein kinases (1,2)

Type I inhibitors bind to the active (DFG-in) conformation of the kinase, occupying the ATP-binding pocket in a manner that competes directly with ATP. Because the ATP-binding site is highly conserved across the kinome, Type I inhibitors can exhibit broad-spectrum activity, although selective Type I inhibitors have also been developed through exploitation of residue-level differences in the binding pocket. Representative examples include erlotinib (EGFR), crizotinib (ALK/MET), and brigatinib (ALK) (1,3).

Type II inhibitors bind to the inactive (DFG-out) conformation of the kinase, extending beyond the ATP-binding site into an adjacent hydrophobic allosteric pocket that becomes accessible only when the DFG motif is flipped outward. This dual-site occupancy typically confers greater selectivity compared to Type I inhibitors, as the allosteric pocket is less conserved across kinases. Representative examples include imatinib (ABL), sorafenib (RAF/VEGFR), and ponatinib (ABL/DDR1) (2,4).

Some kinase inhibitors can adopt both Type I and Type II binding modes depending on the target kinase and its conformational state. For example, sunitinib has been reported to bind in a Type I mode to certain kinases while adopting a Type II orientation in others (5,6).

In the case study presented in this work, Brigatinib was selected as a representative Type I inhibitor (b), Ponatinib as a Type II inhibitor (4,7), and Sunitinib as an inhibitor capable of exhibiting both Type I and Type II binding characteristics (5).

Supplementary Note S3. Test Data Description

Five reference datasets were constructed for model validation. All datasets were compiled based on the DrugBank database (1) accessed on September 25, 2025, with kinase targets mapped to DeepKinomeWeb entries using UniProt accession identifiers. The detailed definitions of each dataset are as follows.

Approved on-target data. Among drugs with "Approved" status in DrugBank, 692 drugs targeting kinases registered in the DeepKinomeWeb kinase panel were initially selected. Of these, 688 drugs that successfully passed the DeepKinome processing pipeline were retained. Predicted binding affinity (% inhibition) data were then filtered to include only drug–kinase pairs matching the annotated drug–target relationships in DrugBank, yielding 422 approved on-target pairs used in the analysis.

For the **off-target data**, drugs targeting kinases listed in DeepKinomeWeb were filtered from DrugBank (8). Subsequently, predicted binding affinity (% inhibition) data between these drugs and off-target kinases within DeepKinomeWeb were included.

For **non-kinase data**, a kinase list was compiled using KinMap (9). Drugs that do not target these kinases were extracted from DrugBank, and their predicted binding affinity (% inhibition) data against all kinases in DeepKinomeWeb were included.

Regarding **general compound data**, organic compounds were randomly sampled and queried from the entire CID range of the PubChem database (10), excluding substances listed in DrugBank. This selection was limited to compounds satisfying the criteria for PaDEL calculations within the DeepKinome pipeline. For the queries that successfully passed the final pipeline, predicted binding affinity (% inhibition) data against all kinases listed in DeepKinome were included.

For **unapproved on-target data**, drugs were filtered based on their unapproved status. The predicted binding affinity (% inhibition) data for these drugs against all kinases in DeepKinomeWeb were included.

Supplementary Note S4. Interpretation of Divergent Selectivity Patterns in FDA-Approved Kinase Inhibitors

In this study, we employed two complementary metrics, SI_{mean} and SI_{worst}, to evaluate kinase inhibitor selectivity. As shown in **Supplementary Fig. S1**, FDA-approved on-target drugs exhibited higher SI_{mean} values compared to off-target or general compound groups, consistent with the expectation that approved drugs generally possess favorable selectivity profiles across the kinome.

However, as illustrated in **Supplementary Fig. S2**, the same approved drugs showed lower S_{Iworst} values (indicating lower selectivity toward the single strongest off-target) compared to the control groups. This apparent divergence reflects the fundamental biological differences between optimized drugs and random compounds, as well as the mathematical properties of the metrics.

1. Intentional Polypharmacology of Kinase Inhibitors Many FDA-approved kinase inhibitors are designed or known to act as multi-target agents (polypharmacology) to enhance therapeutic efficacy or overcome resistance. (11,12) Consequently, even highly effective drugs often possess at least one distinct off-target with high binding affinity (low percent inhibition) (13). Since SI_{worst} is determined solely by the single strongest off-target interaction ($\min(\mathrm{I}_{\mathrm{off-target}})$), the presence of a potent secondary target results in a low SI_{worst} score. This indicates that the DeepKinome model correctly captures the specific off-target liabilities known to exist in approved therapeutics.

2. Sensitivity to Extreme Values vs. Global Distribution SI_{mean} incorporates the geometric mean of all off-target interactions, thereby reflecting the global selectivity landscape (14). In contrast, SI_{worst} is an extreme-value metric. For the "Off-target" or "General Compound" groups, the majority of compounds may interact weakly with both the target and off-targets. When both on-target and off-target affinities are weak (high percent inhibition values), the resulting ratio (SI) can act as a noise factor or appear artificially high due to the lack of potent interaction (15). Conversely, approved drugs exhibit strong on-target binding (very low percent inhibition), so even a moderately strong off-target interaction significantly impacts the ratio.

Conclusion Therefore, the observation that approved drugs have lower SI_{worst} values than reference groups is not an artifact but a reflection of the "narrow polyselectivity" characteristic of many kinase inhibitors. The combination of high SI_{mean} (global selectivity) and variable SI_{worst} (specific off-target risk) provided by DeepKinomeWeb offers a more granular and realistic perspective for prioritization than single metrics alone.

Supplementary Table S1. Comparison of DeepKinomeWeb with existing computational platforms for kinase inhibitor analysis and drug–target interaction prediction

Platform	*Category*	*Primary Function & Focus*	*Targeting Approach*	*Quantitative Prediction*	*Panel-level Selectivity*	*Integrated Analysis*
DeepKinomeWeb *(16)*	**Predictive Engine**	Kinase inhibitor screening & selectivity profiling	**Ligand-centric** (Compound → Panel)	**Yes** (% inhibition)	**Yes** (229 kinases)	**Yes** (Docking + ADMET)
Dr. Kinase *(17)*	DTI Prediction Server	Impact of kinase mutations on drug resistance	Target-centric (Mutation → Drug)	No (Resistance classification)	No	No
KinaseMD *(18)*	Database	Kinase mutations and drug resistance data retrieval	Target-centric	No (Data retrieval only)	No	No
SuperPred 3.0 *(19)*	DTI Prediction Server	Anatomical Therapeutic Chemical (ATC) code & target prediction	Ligand-centric	No (Probability/Classification)	No	No
E3Docker *(20)*	DTI Prediction Server	Docking server for E3 ligase binder discovery	Target-centric	No (Docking score)	No	Yes (Docking only)
ADMETlab 3.0 (21) / ProTox-3.0 *(22)*	Property Predictors	Pharmacokinetics, ADMET, and toxicity prediction	Ligand-centric	No (Target binding affinity)	No	Yes (ADMET/Toxicity only)
BindingDB *(23)*	Bioactivity Resources	Experimental bioactivity data search and retrieval	Target/Ligand-centric	No (Data retrieval only)	No	No
AlphaFold *(24)*	Structure Predictors	3D structure and protein-ligand pose prediction	Target-centric	No (Structural pose/Confidence)	No	Yes (Structural only)

Supplementary Table S2. Server 229 gene list and corresponding UniProt accessions

Gene symbol	UniProt accession	Gene symbol	UniProt accession
BMR1B	O00238	EPHB3	P54753
PLK4	O00444	EPHA5	P54756
STK25	O00506	EPHB4	P54760
MP2K7	O14733	EPHB1	P54762
CHK1	O14757	CSK21	P68400
CSKP	O14936	PI42B	P78356
AURKA	O14965	KC1G2	P78368
GAK	O14976	M3K9	P80192
DCLK1	O15075	CDKL1	Q00532
MK13	O15264	CDK5	Q00535
PDPK1	O15530	CDK16	Q00536
DAPK3	O43293	KPCE	Q02156
M3K7	O43318	MP2K1	Q02750
RIPK2	O43353	TIE2	Q02763
JAK2	O60674	KPCT	Q04759
ROCK2	O75116	ACVR1	Q04771
ULK1	O75385	RON	Q04912
ERN1	O75460	FAK1	Q05397
KS6A5	O75582	BTK	Q06187
STK16	O75716	ACK1	Q07912
PAK3	O75914	DDR1	Q08345
CDKL5	O76039	ITK	Q08881
ST17B	O94768	M3K12	Q12852
STK10	O94804	MERTK	Q12866
OXSR1	O95747	STK4	Q13043
PAK4	O96013	AAPK1	Q13131
CHK2	O96017	PAK1	Q13153
ABL1	P00519	MK07	Q13164
EGFR	P00533	STK3	Q13188
RAF1	P04049	PRP4K	Q13523
ERBB2	P04626	RIPK1	Q13546
NTRK1	P04629	KCC2B	Q13554
INSR	P06213	KCC2G	Q13555
LCK	P06239	KCC2D	Q13557
FYN	P06241	DYR1A	Q13627
CSF1R	P07333	AVR2B	Q13705
LYN	P07948	BMPR2	Q13873
RET	P07949	PTK6	Q13882
IGF1R	P08069	KGP1	Q13976
MET	P08581	CDK13	Q14004
HCK	P08631	KCC1A	Q14012

Gene symbol	UniProt accession	Gene symbol	UniProt accession
ROS1	P08922	FAK2	Q14289
undefined	P0A5S4	MELK	Q14680
WEE2	P0C1S8	STK38	Q15208
KIT	P10721	ERBB4	Q15303
PIM1	P11309	EPHA7	Q15375
FGFR1	P11362	KS6A1	Q15418
CDK4	P11802	MK11	Q15759
SRC	P12931	STK11	Q15831
BRAF	P15056	NTRK3	Q16288
PHKG2	P15735	PKN1	Q16512
KAPCA	P17612	PKN2	Q16513
VGFR1	P17948	KCC4	Q16566
E2AK2	P19525	NTRK2	Q16620
CSK22	P19784	MK06	Q16659
FGFR2	P21802	DDR2	Q16832
ERBB3	P21860	AAK1	Q2M2I8
FGFR4	P22455	TNI3K	Q59H18
FGFR3	P22607	LRRK2	Q5S007
KS6B1	P23443	ULK3	Q6PHR2
JAK1	P23458	MARK2	Q7KZI7
KPCL	P24723	VRK2	Q86Y07
CDK2	P24941	MYLK4	Q86YV6
AVR2A	P27037	KCC1D	Q8IU85
MK03	P27361	M4K3	Q8IVH8
MARK3	P27448	CDKL3	Q8IVW4
MK01	P28482	ULK2	Q8IYT8
EPHA2	P29317	KKCC1	Q8N5S9
EPHA3	P29320	COQ8A	Q8NI60
EPHA8	P29322	PI42C	Q8TBX8
EPHB2	P29323	NEK7	Q8TDX7
TYK2	P29597	ST32A	Q8WU08
WEE1	P30291	DYRK2	Q92630
UFO	P30530	CDKL2	Q92772
AKT1	P31749	M4K1	Q92918
AKT2	P31751	AURKB	Q96GD4
TTK	P33981	MARK4	Q96L34
VGFR2	P35968	KCC1G	Q96NX5
MP2K2	P36507	NEK1	Q96PY6
FLT3	P36888	KKCC2	Q96RR4
TGFR1	P36897	PMYT1	Q99640
ACVL1	P37023	M3K5	Q99683
TGFR2	P37173	RIOK1	Q9BRS2

Gene symbol	UniProt accession	Gene symbol	UniProt accession
CSK	P41240	MKNK1	Q9BUB5
KPCI	P41743	RIOK2	Q9BVS4
ABL2	P42684	SLK	Q9H2G2
ZAP70	P43403	TAOK3	Q9H2K8
KSYK	P43405	HIPK2	Q9H2X6
MK08	P45983	PLK3	Q9H4B4
MK09	P45984	CLK4	Q9HAZ1
MP2K4	P45985	MKNK2	Q9HBH9
KC1A	P48729	KC1G1	Q9HCP0
KC1D	P48730	PAK6	Q9NQU5
MAPK2	P49137	SNRK	Q9NRH2
CDK8	P49336	BMP2K	Q9NSY1
KC1E	P49674	IRAK4	Q9NWZ3
CLK1	P49759	M3K20	Q9NYL2
CLK2	P49760	PLK2	Q9NYY3
CLK3	P49761	MARK1	Q9P0L2
GSK3B	P49841	PIM2	Q9P1W9
CDK7	P50613	PAK5	Q9P286
CDK9	P50750	STK26	Q9P289
IRAK1	P51617	E2AK4	Q9P2K8
KS6A3	P51812	PI4KB	Q9UBF8
BMX	P51813	TBK1	Q9UHD2
NEK2	P51955	DAPK2	Q9UIK4
JAK3	P52333	KS6A6	Q9UK32
MP2K6	P52564	TNIK	Q9UKE5
PLK1	P53350	ALK	Q9UM73
DAPK1	P53355	AURKC	Q9UQB9
LIMK1	P53667	KCC2A	Q9UQM7
LIMK2	P53671	IRAK3	Q9Y616
MK12	P53778	STK24	Q9Y6E0
MK10	P53779	KC1G3	Q9Y6M4
AAPK2	P54646		

Reference

1. Roskoski, R., Jr. (2016) Classification of small molecule protein kinase inhibitors based upon the structures of their drug-enzyme complexes. *Pharmacol Res*, **103**, 26-48.

http://www.ncbi.nlm.nih.gov/pubmed/26529477

http://dx.doi.org/10.1016/j.phrs.2015.10.021

2. Zhao, Z., Wu, H., Wang, L., Liu, Y., Knapp, S., Liu, Q. and Gray, N.S. (2014) Exploration of type II binding mode: A privileged approach for kinase inhibitor focused drug discovery? *ACS Chem Biol*, **9**, 1230-1241.

http://www.ncbi.nlm.nih.gov/pubmed/24730530

http://dx.doi.org/10.1021/cb500129t

3. Huang, W.S., Liu, S., Zou, D., Thomas, M., Wang, Y., Zhou, T., Romero, J., Kohlmann, A., Li, F., Qi, J. *et al.* (2016) Discovery of Brigatinib (AP26113), a Phosphine Oxide-Containing, Potent, Orally Active Inhibitor of Anaplastic Lymphoma Kinase. *J Med Chem*, **59**, 4948-4964.

http://www.ncbi.nlm.nih.gov/pubmed/27144831

http://dx.doi.org/10.1021/acs.jmedchem.6b00306

4. O'Hare, T., Shakespeare, W.C., Zhu, X., Eide, C.A., Rivera, V.M., Wang, F., Adrian, L.T., Zhou, T., Huang, W.S., Xu, Q. *et al.* (2009) AP24534, a pan-BCR-ABL inhibitor for chronic myeloid leukemia, potently inhibits the T315I mutant and overcomes mutation-based resistance. *Cancer Cell*, **16**, 401-412.

http://www.ncbi.nlm.nih.gov/pubmed/19878872

http://dx.doi.org/10.1016/j.ccr.2009.09.028

http://www.ncbi.nlm.nih.gov/pmc/articles/PMC2804470

5. Faivre, S., Demetri, G., Sargent, W. and Raymond, E. (2007) Molecular basis for sunitinib efficacy and future clinical development. *Nature reviews. Drug discovery*, **6**, 734-745.

http://www.ncbi.nlm.nih.gov/pubmed/17690708

http://dx.doi.org/10.1038/nrd2380

6. Papaetis, G.S. and Syrigos, K.N. (2009) Sunitinib: a multitargeted receptor tyrosine kinase inhibitor in the era of molecular cancer therapies. *BioDrugs*, **23**, 377-389.

http://www.ncbi.nlm.nih.gov/pubmed/19894779

http://dx.doi.org/10.2165/11318860-000000000-00000

7. Wang, X., DeFilippis, R.A., Yan, W., Shah, N.P. and Li, H.Y. (2024) Overcoming Secondary Mutations of Type II Kinase Inhibitors. *J Med Chem*, **67**, 9776-9788.

http://www.ncbi.nlm.nih.gov/pubmed/38837951

http://dx.doi.org/10.1021/acs.jmedchem.3c01629

http://www.ncbi.nlm.nih.gov/pmc/articles/PMC11586107

8. Knox, C., Wilson, M., Klinger, C.M., Franklin, M., Oler, E., Wilson, A., Pon, A., Cox, J., Chin, N.E.L., Strawbridge, S.A. *et al.* (2024) DrugBank 6.0: the DrugBank Knowledgebase for 2024. *Nucleic acids research*, **52**, D1265-D1275.

http://www.ncbi.nlm.nih.gov/pubmed/37953279

http://dx.doi.org/10.1093/nar/gkad976

http://www.ncbi.nlm.nih.gov/pmc/articles/PMC10767804

9. Eid, S., Turk, S., Volkamer, A., Rippmann, F. and Fulle, S. (2017) KinMap: a web-based tool for interactive navigation through human kinome data. *BMC bioinformatics*, **18**, 16.

http://www.ncbi.nlm.nih.gov/pubmed/28056780

http://dx.doi.org/10.1186/s12859-016-1433-7

http://www.ncbi.nlm.nih.gov/pmc/articles/PMC5217312

10. Kim, S., Chen, J., Cheng, T., Gindulyte, A., He, J., He, S., Li, Q., Shoemaker, B.A., Thiessen, P.A., Yu, B. *et al.* (2025) PubChem 2025 update. *Nucleic acids research*, **53**, D1516-D1525.

http://www.ncbi.nlm.nih.gov/pubmed/39558165

http://dx.doi.org/10.1093/nar/gkae1059

http://www.ncbi.nlm.nih.gov/pmc/articles/PMC11701573

11. Hopkins, A.L. (2008) Network pharmacology: the next paradigm in drug discovery. *Nature chemical biology*, **4**, 682-690.

http://www.ncbi.nlm.nih.gov/pubmed/18936753

http://dx.doi.org/10.1038/nchembio.118

12. Ferguson, F.M. and Gray, N.S. (2018) Kinase inhibitors: the road ahead. *Nature reviews. Drug discovery*, **17**, 353-377.

http://www.ncbi.nlm.nih.gov/pubmed/29545548

http://dx.doi.org/10.1038/nrd.2018.21

13. Klaeger, S., Heinzlmeir, S., Wilhelm, M., Polzer, H., Vick, B., Koenig, P.A., Reinecke, M., Ruprecht, B., Petzoldt, S., Meng, C. *et al.* (2017) The target landscape of clinical kinase drugs. *Science (New York, N.Y.)*, **358**.

http://www.ncbi.nlm.nih.gov/pubmed/29191878

http://dx.doi.org/10.1126/science.aan4368

http://www.ncbi.nlm.nih.gov/pmc/articles/PMC6542668

14. Davis, M.I., Hunt, J.P., Herrgard, S., Ciceri, P., Wodicka, L.M., Pallares, G., Hocker, M., Treiber, D.K. and Zarrinkar, P.P. (2011) Comprehensive analysis of kinase inhibitor selectivity. *Nature biotechnology*, **29**, 1046-1051.

http://www.ncbi.nlm.nih.gov/pubmed/22037378

http://dx.doi.org/10.1038/nbt.1990

15. Karaman, M.W., Herrgard, S., Treiber, D.K., Gallant, P., Atteridge, C.E., Campbell, B.T., Chan, K.W., Ciceri, P., Davis, M.I., Edeen, P.T. *et al.* (2008) A quantitative analysis of kinase inhibitor selectivity. *Nature biotechnology*, **26**, 127-132.

http://www.ncbi.nlm.nih.gov/pubmed/18183025

http://dx.doi.org/10.1038/nbt1358

16. Lee, Y., Eun, J., Lee, J. and Nam, S. (2025) DeepKinome: quantitative prediction of kinase binding affinity by a compound using deep learning based regression model. *Front Mol Biosci*, **12**, 1698891.

http://www.ncbi.nlm.nih.gov/pubmed/41415030

http://dx.doi.org/10.3389/fmolb.2025.1698891

http://www.ncbi.nlm.nih.gov/pmc/articles/PMC12709132

17. Lin, S., Tu, C., Hu, R., Wang, H., Dong, Z., Luo, H., Kuang, L., Wang, T., Wang, L., Zhao, Z. *et al.* (2025) Dr. Kinase: predicting the drug-resistance hotspots of protein kinases. *Nucleic acids research*, **53**, W258-w265.

http://www.ncbi.nlm.nih.gov/pubmed/40308214

http://dx.doi.org/10.1093/nar/gkaf366

http://www.ncbi.nlm.nih.gov/pmc/articles/PMC12230729

18. Hu, R., Xu, H., Jia, P. and Zhao, Z. (2021) KinaseMD: kinase mutations and drug response database. *Nucleic acids research*, **49**, D552-d561.

http://www.ncbi.nlm.nih.gov/pubmed/33137204

http://dx.doi.org/10.1093/nar/gkaa945

http://www.ncbi.nlm.nih.gov/pmc/articles/PMC7779064

19. Gallo, K., Goede, A., Preissner, R. and Gohlke, B.O. (2022) SuperPred 3.0: drug classification and target prediction-a machine learning approach. *Nucleic acids research*, **50**, W726-w731.

http://www.ncbi.nlm.nih.gov/pubmed/35524552

http://dx.doi.org/10.1093/nar/gkac297

http://www.ncbi.nlm.nih.gov/pmc/articles/PMC9252837

20. Yan, K., He, W., Pang, M., Lu, X., Chen, Z., Piao, L., Zhang, H., Wang, Y., Chang, S. and Kong, R. (2025) E3Docker: a docking server for potential E3 binder discovery. *Nucleic acids research*, **53**, W266-w272.

http://www.ncbi.nlm.nih.gov/pubmed/40337923

http://dx.doi.org/10.1093/nar/gkaf391

http://www.ncbi.nlm.nih.gov/pmc/articles/PMC12230658

21. Fu, L., Shi, S., Yi, J., Wang, N., He, Y., Wu, Z., Peng, J., Deng, Y., Wang, W., Wu, C. *et al.* (2024) ADMETlab 3.0: an updated comprehensive online ADMET prediction platform enhanced with broader coverage, improved performance, API functionality and decision support. *Nucleic acids research*, **52**, W422-w431.

http://www.ncbi.nlm.nih.gov/pubmed/38572755

http://dx.doi.org/10.1093/nar/gkae236

http://www.ncbi.nlm.nih.gov/pmc/articles/PMC11223840

22. Banerjee, P., Kemmler, E., Dunkel, M. and Preissner, R. (2024) ProTox 3.0: a webserver for the prediction of toxicity of chemicals. *Nucleic acids research*, **52**, W513-w520.

http://www.ncbi.nlm.nih.gov/pubmed/38647086

http://dx.doi.org/10.1093/nar/gkae303

http://www.ncbi.nlm.nih.gov/pmc/articles/PMC11223834

23. Gilson, M.K., Liu, T., Baitaluk, M., Nicola, G., Hwang, L. and Chong, J. (2016) BindingDB in 2015: A public database for medicinal chemistry, computational chemistry and systems pharmacology. *Nucleic acids research*, **44**, D1045-1053.

http://www.ncbi.nlm.nih.gov/pubmed/26481362

http://dx.doi.org/10.1093/nar/gkv1072

http://www.ncbi.nlm.nih.gov/pmc/articles/PMC4702793

24. Abramson, J., Adler, J., Dunger, J., Evans, R., Green, T., Pritzel, A., Ronneberger, O., Willmore, L., Ballard, A.J., Bambrick, J. *et al.* (2024) Accurate structure prediction of biomolecular interactions with AlphaFold 3. *Nature*, **630**, 493-500.

http://www.ncbi.nlm.nih.gov/pubmed/38718835

http://dx.doi.org/10.1038/s41586-024-07487-w

http://www.ncbi.nlm.nih.gov/pmc/articles/PMC11168924
